# T Cell Lymphoma and Leukemia in Severe Combined Immunodeficiency Pigs following Bone Marrow Transplantation: A Case Report

**DOI:** 10.3389/fimmu.2017.00813

**Published:** 2017-07-12

**Authors:** Ellis J. Powell, Jared Graham, N. M. Ellinwood, Jesse Hostetter, Michael Yaeger, Chak-Sum Ho, Lynden Gault, Veronica Norlin, Elizabeth N. Snella, Jackie Jens, Emily H. Waide, Adeline N. Boettcher, Maureen Kerrigan, Raymond R. R. Rowland, Jason W. Ross, Jack C. M. Dekkers, Christopher K. Tuggle

**Affiliations:** ^1^Department of Animal Science, Iowa State University, Ames, IA, United States; ^2^Department of Veterinary Pathology Science, Iowa State University, Ames, IA, United States; ^3^Gift of Life Michigan, Ann Arbor, MI, United States; ^4^Kansas State University, Manhattan, KS, United States

**Keywords:** severe combined immunodeficiency, Artemis, lymphoma, leukemia, pig, bone marrow transplantation

## Abstract

After the discovery of naturally occurring severe combined immunodeficiency (SCID) within a selection line of pigs at Iowa State University, we found two causative mutations in the Artemis gene: haplotype 12 (ART12) and haplotype 16 (ART16). Bone marrow transplants (BMTs) were performed to create genetically SCID and phenotypically immunocompetent breeding animals to establish a SCID colony for further characterization and research utilization. Of nine original BMT transfer recipients, only four achieved successful engraftment. At approximately 11 months of age, both animals homozygous for the ART16 mutation were diagnosed with T cell lymphoma. One of these ART16/ART16 recipients was a male who received a transplant from a female sibling; the tumors in this recipient consist primarily of Y chromosome-positive cells. The other ART16/ART16 animal also presented with leukemia in addition to T cell lymphoma, while one of the ART12/ART16 compound heterozygote recipients presented with a nephroblastoma at a similar age. Human Artemis SCID patients have reported cases of lymphoma associated with a “leaky” Artemis phenotype. The naturally occurring Artemis SCID pig offers a large animal model more similar to human SCID patients and may offer a naturally occurring cancer model and provides a valuable platform for therapy development.

## Case Presentation

After the discovery of the Severe Combined Immunodeficiency (SCID) phenotype in pigs at Iowa State University (ISU), the phenotype’s genetic cause was determined to be two independent mutations segregating from independent founders. To further characterize the SCID pig and to maintain a research population, bone marrow transplantations (BMTs) were performed on select individuals. Interestingly, of four successful transplants, three developed hematological malignancies with possible implications from their different genetic background as described herein.

Matings between heterozygous dams and a sire produced litters from which 25% of piglets had the SCID phenotype and required BMT to rescue. This would result in genetically SCID animals, but phenotypically competent immune systems. If fertile, these animals would allow SCID to carrier matings producing litters of 50% SCID piglets, which is advantageous for experimental and characterization work. After farrowing, tissues samples were tested for Artemis genotype and MHC haplotype. Between four litters across two rounds of transplantation, nine SCID piglets were transplanted with unfractionated bone marrow from 100% matched MHC, opposite sex donors, by intravenous injection. Of the nine recipients, five were euthanized due to degrading health within the first 3 months post transplantation.

**Figure 1:**
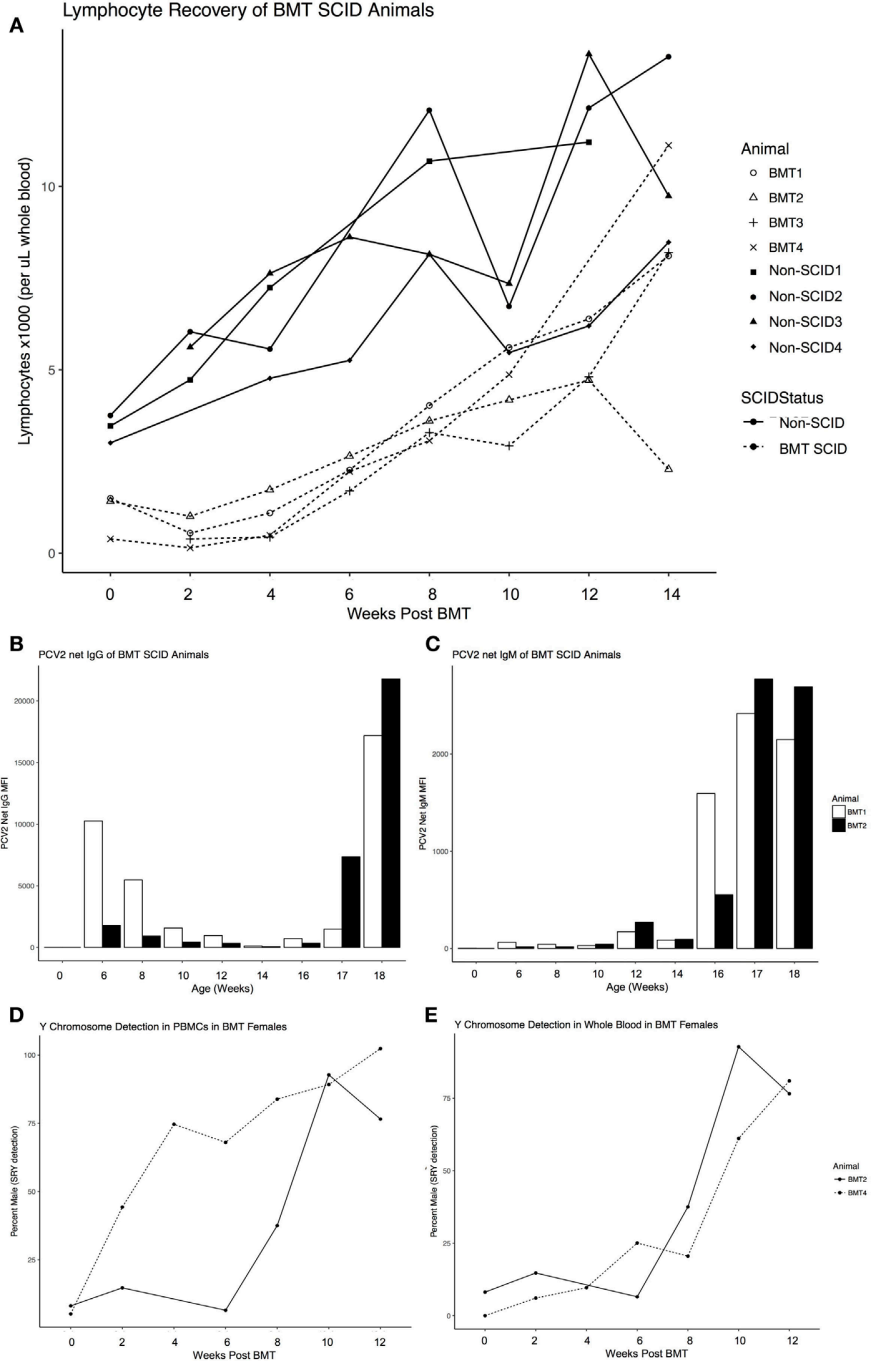
Engraftment parameters of Severe Combined Immunodeficiency (SCID) pigs after opposite sex, 100% MHC matched donor bone marrow transplantation. **(A)** Recovery of lymphocyte numbers to within a normal range of BMT SCID pigs (dashed line) compared to non-SCID littermates (solid line) monitored by complete blood counts ever 2 weeks after transplant. **(B)** Antibody response to vaccination for Circumvent (against circovirus) for both ART16/ART16 pigs (BMT1, BMT2) for total immunoglobulin (Ig)G and **(C)** IgM. Only applicable to female recipients of male donor cells, pig SRY gene expression (detection of Y Chromosome) was determined as a percentage of sample from **(D)** isolated PBMCs and **(E)** whole blood from BMT2, BMT4. Figure created with ggplot2 (32).

The four surviving transplant recipients, consisting of two ART16/ART16 pigs from the first round of transplants and two ART12/ART16 pigs from the second, achieved successful engraftment. We monitored lymphocyte numbers, production of specific antibodies in response to vaccination, and presence of Y chromosome as engraftment parameters. Lymphocyte numbers were evaluated using complete blood counts (CBCs) every 2 weeks after BMT and found that lymphocytes reached a near normal level around approximately 8–10 weeks post BMT (Figure 1A). As the capability to mount an antibody response to vaccination would be a strong indicator of engraftment success, both ART16/ART16 animals were injected with killed porcine circovirus type 2 (PCV2) vaccine 10–11 weeks post BMT and again 14–15 weeks post BMT. Serum was analyzed every 2 weeks for immunoglobulin (Ig)G or IgM against PCV2. As seen in Figure 1B for IgG, and in Figure 1C for IgM, both animals mounted successful antibody responses. Of note, in Figure 1B, initial levels of IgG that decreased during the first 12–14 weeks of life are consistent with maternal antibody decay. Finally, because donors were opposite sex to recipients, we measured the amount of *SRY* DNA (Y chromosome) content in the female BMT recipients, which as expected with successful engraftment, increased over time in both peripheral blood mononuclear cells (PBMCs) and whole blood (Figures 1D,E).

Three of the four successful bone marrow transplants were euthanized from 11 to 13 months of age, and the last one at 4 years of age, due to deteriorating health. The significant pathology findings are described below. Descriptions of pathology follow veterinary medicine standards of scoring and grading for neoplasm classification.

**Figure 2:**
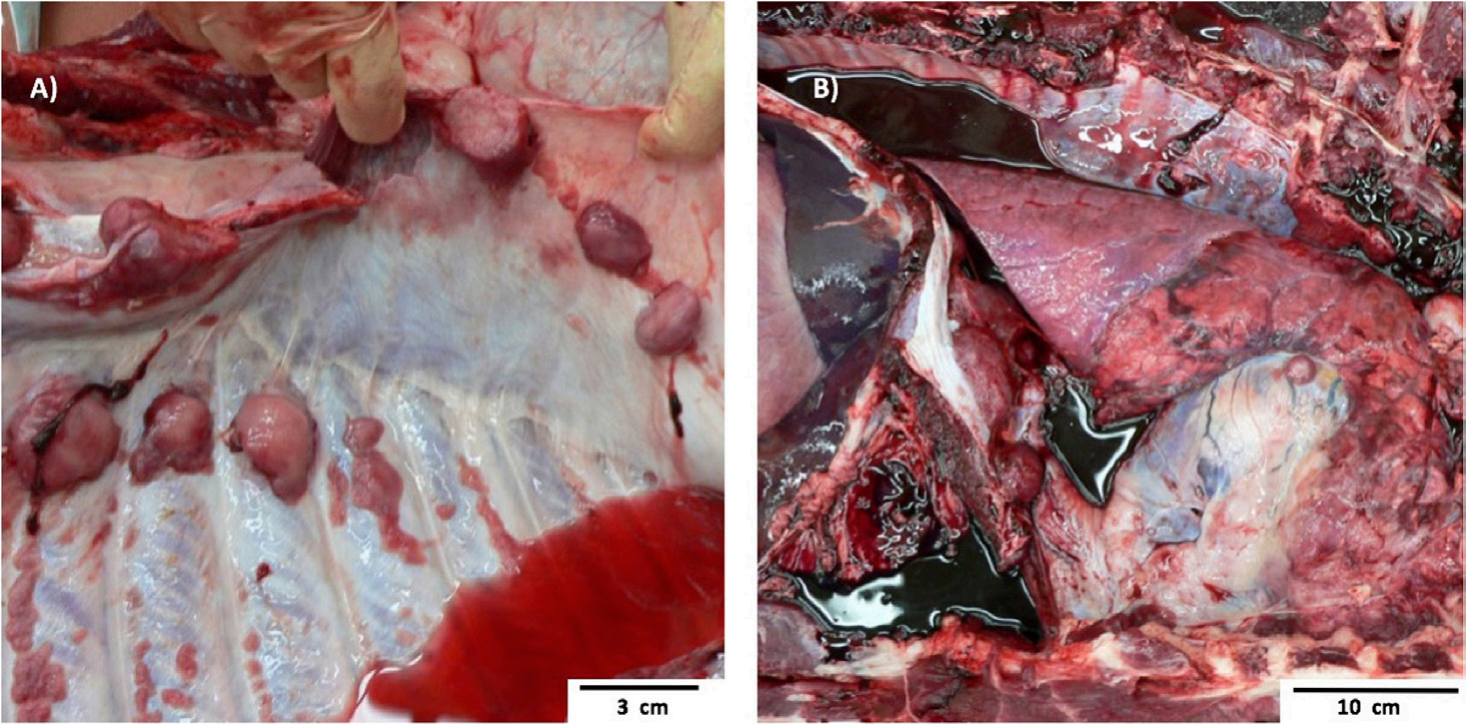
Gross Pathology images of two separate ART16/ART16 pigs (BMT1, BMT2) diagnosed with T cell lymphoma. **(A)** Multiple smooth tan nodules 1–6 cm in diameter in the lung from BMT1 and similar **(B)** tan cranial mediastinal mass of thymic origin from BMT2.

**Figure 3:**
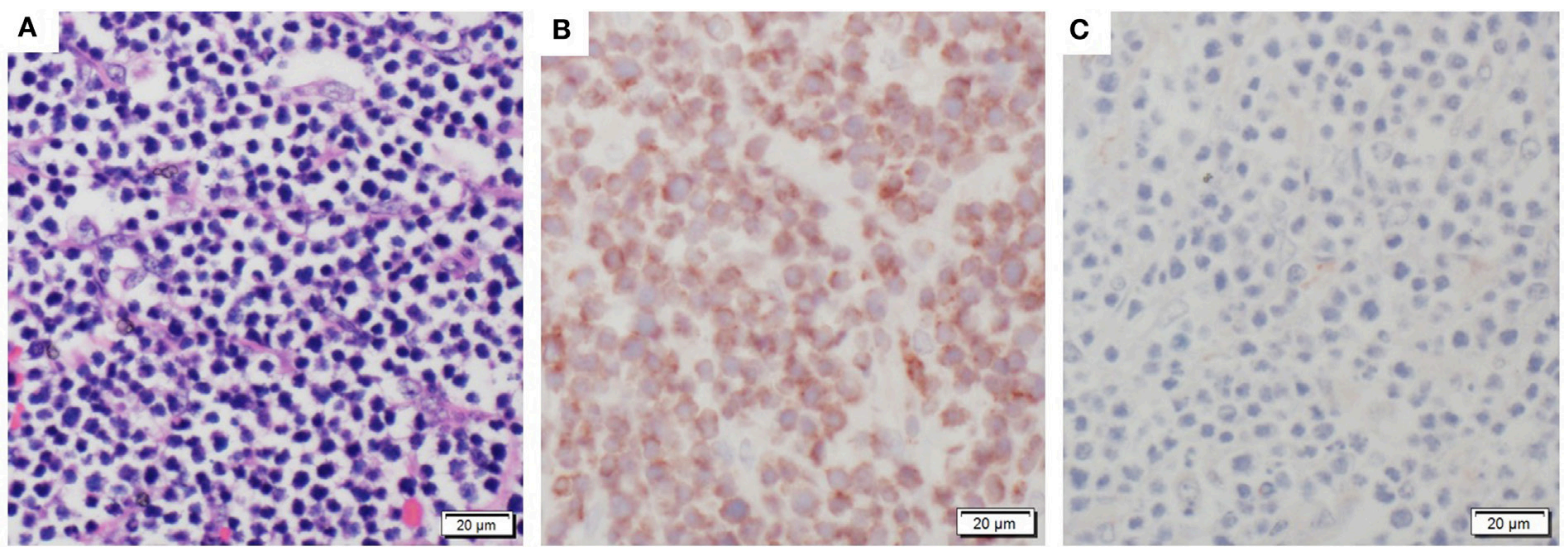
Histologic staining documenting characteristic T cell lymphoma. **(A)** An example of the neoplastic sheets collected from thoracic tumor. **(B)** Stained tumor mass showing CD3+ T cell positive phenotype. **(C)** Stained tumor mass showing CD79− B cell negative tissue staining.

Pig 1 Bone Marrow Transplantation case 1 (BMT1) was a 13-month-old ART16/ART16 male that presented with a thoracic mass. This was a 30-cm tan solid mass located immediately rostral to the heart. This mass had infiltrated the thoracic aorta, cranial mediastinum, and pericardium. Similar masses 1–6 cm in diameter were observed within the lungs, and on the parietal pleural of the chest wall (Figure 2A) and diaphragm. Microscopically these masses were composed of densely packed sheets of small to medium sized lymphocytes (Figure 3A). Immunohistochemistry on the mediastinal mass with CD3 (T cell) and CD79 (B cell) specific antibodies demonstrated that neoplastic cells stained uniformly for CD3 and were negative for CD79 (Figures 3B,C). This staining profile indicates that the neoplastic lymphocytes had a T cell phenotype. A QPCR analysis of the *SRY* gene for three separate 16/16 male tumor samples assaying for the Y chromosome showed that the tumors were derived from cells of the recipient male rather than from female donor bone marrow cells (see Table 1). While the variation in male cell percentage is unclear, we speculate that variable amounts of female donor cells were present in the immune tissue as part of the biopsy, explaining the variation of *SRY* presence from the tumor sections seen in Table 1.

**Table 1:** Y chromosome detection in tumor samples from BMT1.

Tumor sample	Percent male
Neoplastic I	46.20
Neoplastic II	60.24
Neoplastic III	97.68

*Percent male (concentration of *SRY* Y chromosome detection) of various tumor samples of BMT1, the homozygous ART16 male, diagnosed with T cell lymphoma*.

Pig 2 (BMT2), an 11-month-old ART16/ART16 female had a 5-cm tan cranial mediastinal mass (Figure 2B). Microscopically the mass was composed of densely packed uniform sheets of small to medium sized neoplastic lymphocytes similar to those seen in Figure 3A for BMT1. Neoplastic lymphocytes had scant to moderate amounts of cytoplasm with a small round nucleus. The mitotic rate was low. Neoplastic lymphocytes were also identified microscopically in the bone marrow, lymph node, thymus, lung, colon, and small intestine. Antemortem CBCs demonstrated abnormally high numbers of circulating lymphocytes suggesting extension of neoplastic lymphocytes into circulation. Neoplastic lymphocytes were diffusely positive for CD3 and negative for CD79 as seen in Figures 3B,C. These findings are consistent with a diagnosis of T cell lymphoma. Based on the morphology and phenotype of neoplastic lymphocytes and their distribution in tissues and blood a diagnosis of chronic lymphocytic leukemia was made, though multicentric T cell lymphoma with a leukemic blood profile could not be definitively excluded.

Pig 3 (BMT3), ART12/ART16 male lived until 4 years of age. He had severe distention of the pericardium by clear fluid and there was ~2 l of clear fluid in the abdominal cavity. The epicardial surface was covered in a friable dark brown exudate. Microscopically this exudate consisted of immature fibrous connective tissue that was infiltrated by macrophages and lymphocytes. In the subendocardium, there was myocyte loss and fibrosis. Additional microscopic changes were present in the liver where there was congestion of centrilobular sinusoids and necrosis of centrilobular hepatic cords. Together these gross and microscopic changes indicate right-sided heart failure. An underlying etiology was not identified. However, bacterial agents such as *Mycoplasma hyorhinis* and *Haemophilus parasuis* would be possible. No evidence of a neoplastic process was identified in BMT3.

Pig 4 (BMT4) was a ART12/ART16 female 12 months of age that had a 20 cm × 30 cm irregular mass in the retroperitoneal space which compressed the right kidney. The right kidney, caudal vena cava, and a loop of duodenum were attached to the mass *via* firm fibrous adhesions. Microscopically the mass was composed of a population of neoplastic epithelial cells arranged into irregular tubular structure separated by densely packed streams of spindle shaped cells. The mitotic rate was high in both the epithelial and mesenchymal populations with 1–2 mitoses per 400× field. The location of the neoplasm and its gross and microscopic features were consistent with a diagnosis of nephroblastoma.

BMT1, the first male, was fertile and a number of doses of semen were collected, which have been used subsequently to create litters. The second male, BMT3, lived 4 years as a successful breeding boar, having created over eight litters in that time, and for which semen was collected and frozen and is being used for additional litters.

## Background

Severe Combined Immunodeficiency is defined as the lack or impairment of the adaptive immune system and can be caused by over 30 gene defects (1). In addition to humans, SCID is naturally occurring in the horse (2), dog (3), mouse (4), and as of 2012, the pig (5). This SCID pig was serendipitously discovered during a virus challenge study in collaboration with Kansas State University with ISU Yorkshire pigs that had been divergently selected for feed efficiency (6). Four pigs died unexpectedly and during routine necropsy presented with symptoms consistent with SCID: virtually absent of antibodies, lacked or had an unusually small thymus, and presented additional underdeveloped immune tissues (5). A genome-wide association study identified a strong correlation between the SCID phenotype and a candidate peak on chromosome 10, which contains the Artemis (*DCLRE1C*) gene (7). During Variable (V), Diversity (D), and Joining (J) segment recombination, the endonuclease Artemis cleaves open the hairpin loop created by recombination activating genes (*RAG*)1 and *RAG*2 (1, 8). Thus, individuals with severe Artemis defects cannot create mature lymphocytes with B cell receptors or T cell receptors, causing an Artemis SCID phenotype (8). Although genetically engineered SCID pigs exist for modified interleukin-2 receptor γ gene (IL2RG) (9, 10) as well as for *RAG*1 and/or 2 (11, 12), the ISU SCID pig is the only naturally occurring SCID pig. Consistent with human SCID Artemis patients, the ISU SCID pig is radiosensitive, and has a T cell-, B cell-, natural killer (NK) cell^+^ phenotype (7, 13, 14).

Interestingly, two separate point mutations within the Artemis gene are responsible for the SCID phenotype (7). Termed ART12 and ART16, these alleles travel in a Mendelian recessive mode of inheritance in either homozygous (ART12/ART12, ART16/ART16) or compound heterozygous (ART12/ART16) states (7). The ART12 allele has a point mutation in exon 10 that creates a frame shift and premature stop codon, which results in less than 40% of the Artemis protein produced: thus animals carrying homozygous ART12 mutations are expected to have no Artemis activity (7). The ART16 mutation abrogates a 5′ splice site signal sequence in intron 8 that alters a splice signal sequence and causes the deletion of 141 nucleotides and exon 8. As this deletion does not compromise the reading frame, it is possible the transcripts could result in a partially functional Artemis protein (7). While Artemis VDJ recombination or DNA repair activity assays for a collection of human Artemis patient allele-encoded proteins have been performed (15, 16), there are no reported assays involving exon 8-only deletions or missense point mutants. It is interesting that such assays have shown partial activity for point mutants in exon 7 and 9 (16), thus the swine allele with the ART16 mutation affecting exon 8 only may encode a partially active protein. However, transcripts isolated from fibroblasts derived from 16 homozygous pigs show many different structures, with only a minority containing a deleted exon 8 only.

## Discussion

In summary, both ART16/ART16 BMT pigs (BMT1, BMT2) were diagnosed with neoplastic disease of T lymphocytes, one with T cell lymphocytic leukemia and each with T cell lymphoma. The third successful BMT pig was found to have chronic heart failure (BMT3), and a nephroblastoma was found in the last BMT pig, BMT4; both were of the ART12/ART16 heterozygous genotype.

Although the ISU SCID pigs have a T-B-NK^+^ environment, we showed successful bone marrow engraftment increased lymphocytes to within normal ranges, a positive antibody response to vaccination, and in female recipients who received male donor cells, an increase in Y chromosome (SRY gene) detection over time after transplantation. The presence of functional NK cells in the SCID environment has been known to affect engraftment success of BMT procedures as well as the likelihood of Graft versus Host Disease (17). This is an obstacle for human SCID patients that we may be able to model and improve therapies by using the NK^+^ ISU SCID pig.

The ISU SCID pig is well-poised as a biomedical cancer model. The SCID pig does not reject xenotransplants, as growth of human pancreatic carcinoma and human melanoma cells was established in the ears of SCID piglets but not in non-SCID littermates (18). The ability of SCID pigs to allow growth of human cells creates a potential model for investigating stability and efficacy of stem cell-derived therapies for many medical conditions. Such pigs also provide a disease model to study and improve therapies for human SCID patients. One such example would include the improvement of BMTs, which is a common therapy to rescue the SCID phenotype (17). The size and anatomical similarities between pigs and humans compared to classic rodent models also facilitates novel approaches to pharmacology and drug dosing research important to improving human patient therapies. Although several versions of SCID mice have been genetically engineered as biomedical models, the pig is more physiologically similar in anatomy and size to a human than is the mouse (19) in addition to having a more similar “immunome” (20), which may make research therapy progress in pigs more directly applicable to the human patient.

Furthermore, “leaky” behavior of some Artemis defects has been shown to exist in human Artemis patients and can be associated with lymphoma (21). SCID causing mutations that are hypomorphic can sometimes result in low level development of circulating T cells described as leaky SCID (LS) (21), and a LS mouse model has recently been developed for Artemis (22). LS SCID patients are still severely at risk from pathogen threat and often have additional autoimmune complications caused by a lack of appropriate immune signaling of existing T cells (21). Along with increased risk of lymphoma in leaky human Artemis patients (21), Artemis patients are at much higher risk for late-stage infection following BMT procedures and for repeat of such BMT (8). In a 2013 review describing 14 SCID patients affected by a hypomorphic Artemis mutations, three patients presented with lymphoma (associated with Epstein Barr virus), six were successfully rescued by transplantation, and three that received transplants died due to complications (23). It is conceivable that the homozygous ART16 SCID pig could produce a partial Artemis protein demonstrating a leaky phenotype. More characterization and long-term analysis of ART16/ART16 would be necessary to confirm this pathogenesis. Regardless, considering the dearth of lymphoma large animal biomedical models, the naturally occurring SCID pig offers a relevant model for treatment and therapy research.

## Concluding Remarks

The pig is more similar immunologically and physiologically to the human than the mouse. Thus a porcine model without adaptive immunity is valuable for studying immune cell subsets, regenerative medicine, and therapeutic strategies for cancer treatment. The repeated occurrence of cancer within such a limited number of animals and the unique cases of lymphoma and leukemia herein suggest the ART16/ART16 homozygous SCID pig may be a valuable tool to study specific forms of cancer, and potentially model treatments for human lymphoma/leukemia patients.

## Materials and Methods

### Creation of SCID Litters

Carrier × carrier matings were conducted to produce litters expected to contain 25% affected SCID piglets. DNA was isolated from ear or tail tissue after piglet processing and shipped to Gift of Life Michigan for MHC typing. SCID status was determined through PCR testing as described in Waide et al. (7).

### Swine Leukocyte Antigen (SLA) Genotyping

Swine leukocyte antigen genotyping of three class I (*SLA-1, SLA-2, SLA-3*) and three class II (*DRB1, DQB1, DQA*) genes was performed using PCR-based assays with sequence-specific typing primers as previously described (24–26). Modifications were made to the typing primer panels to broaden the allele coverage with the increasing number of SLA alleles. Parental SLA haplotypes and allele-level resolution were assigned based on published data (24, 25, 27–29) and confirmed by inheritance and segregation in the offspring.

### Bone Marrow Preparation

Donor femurs were aseptically removed, washed in ethanol, and moved to a sterile biological safety cabinet. Bone ends were cut to expose the marrow. Using a syringe and needle, marrow was flushed with HBSS (Sigma cat# H6648) into a sterile beaker. Bone marrow particles were aspirated/triturated and spun at 1,500 rpm for 5 min. Cells were resuspended in 100 ml ACK (Lonza No.:10548E) and incubated at RT for 10 min. The marrow suspension was filtered through a cell strainer (Falcon, Cat# 352350). The supernatant was removed and resuspended in 10 ml HBSS and spun at 1,500 rpm for 5 min. The wash was repeated and cell suspension was counted for live cells prior to injection using a hemocytometer.

### Bone Marrow Transplants

BMTs were performed *via* intravenous injection of unfractionated bone marrow cells from 100% MHC matched, opposite sex full or half-sib donors so that sex chromosome assays could be used for tracking donor cells. All available cells from donor piglet were injected into the MHC matched recipient (typically between 1 × 10^9^ and 8 × 10^9^ cells/kg).

### Complete Blood Counts

Complete blood counts were performed on EDTA treated blood samples with an automated cell counter at the Clinical Pathology Laboratory in the College of Veterinary Medicine at ISU.

### Y Chromosome Detection

Y Chromosome presence was determined through PCR detection of the *SRY* gene (RefSeq assembly accession: GCF_000003025.5) in DNA from whole blood, PBMCs, and tissue DNA. Initial primer sequences were from Pomp et al. (30) but were modified to increase efficiency to a final assay utilizing the following primers:

Forward primer 5′-GAGGGCACAGAATTTGCTTC-3′
Reverse primer 5′-GGCTTTCTGTTCCTGAGCAC-3′

Conditions were taken from the Pomp et al. (30) paper. This protocol was modified to increase PCR efficiency by implementing a two-step amplification. There was a 95°C degree ramp up for 5 min followed by 40 cycles of 95°C for 2 min then 62°C for 1 min. For quantitative PCR, a Bio-Rad MyIQ Real Time Thermal Cycler was used with a melting curve to determine PCR product specificity.

### Response to Vaccination

Animals were vaccinated with Circumvent (Merck Animal Health product 103904) against PCV2 10–11 weeks post BMT and again 14–15 weeks post BMT. Serum samples were collected every 2 weeks and analyzed *via* fluorescent microsphere immunoassay for total Ig as well as IgM against PCV2 (31).

### Immunohistochemistry Staining

Unstained 3-µm thick slides were prepared from formalin fixed paraffin embedded tissues. The CD79 (DAKO monoclonal mouse anti-human, B-cell clone HM57) primary antibody was used at a concentration of 200 µg/ml, and the CD3 primary antibody (DAKO monoclonal mouse anti-human, F7.2.38) was used at a concentration of 0.60 µg/ml. CD3 antibody was incubated for 90 min and CD79 for 60 min at room temperature. Appropriate secondary antibodies were used for 15 min with multilink (1.25 mg/ml) HRP (2.5 mg/2 ml) detection system.

## Ethics Statement

All animal work was conducted in accordance with USDA guide to large animal requirements in the Animal Welfare Act and approved by the Institutional Animal Care and Use Committee (IACUC).

## Author Contributions

EP interpreted results, constructed figures, and wrote the initial draft of the manuscript. NE and JR organized and performed BMT transplantations. MY performed necropsies on animals of interest, collected samples, and wrote official pathology reports, and JH wrote the section on pathology findings and edited the manuscript. JG developed and conducted SRY PCR analysis of blood and tumor tissue samples. CH, LG, and VN performed MHC typing of litters and their parents. ES isolated bone marrow from donor animals, and JJ managed piglet anesthesia during BMT. EW genotyped animals for SCID status. AB was involved in concept development and editing of manuscript. MK and RR analyzed blood samples and provided data for response to vaccination of BMT animals. JD is in charge of the SCID colony at L. Christian Swine Breeding farm and edited the manuscript. CT was involved with aspects of design, execution, interpretation of results, and writing of the manuscript.

## Conflict of Interest Statement

The authors declare that the research was conducted in the absence of any commercial or financial relationships that could be construed as a potential conflict of interest. The reviewers, WD and RP, and handling editor declared their shared affiliation and the handling editor states that the process nevertheless met the standards of a fair and objective review.
